# Shifting Positionalities in a Time of COVID-19: The Transnational Public Health Doctor and Ethnographer

**DOI:** 10.3389/fsoc.2021.776968

**Published:** 2021-12-02

**Authors:** Sepeedeh Saleh

**Affiliations:** ^1^ Liverpool School of Tropical Medicine, Liverpool, United Kingdom; ^2^ Malawi-Liverpool-Wellcome Trust Clinical Research Programme, Blantyre, Malawi

**Keywords:** ethnography (methods), positionalities in research, reflexivity, researcher-participant relationship, COVID-19

## Abstract

Ethnographic research is characterised by in-person engagement with individuals and groups within a social setting, usually over an extended timeframe. These elements provide valuable insights which cannot be gained through other forms of research. In addition, such levels of involvement in “the field” create complex, shifting researcher-participant relationships which themselves shape the course of the project and its findings. The COVID-19 pandemic disrupted many research projects, but impacts on ethnographic research, with its emphasis on physical presence in the field and interpersonal relationships, reveals much about these key elements of our praxis.

I discuss how the pandemic influenced the progress of an ethnographic research project, based in Malawi, including consideration of how, as lead for the project, my clinical/“public health” positionalities interacted with relationships in the village and the arrival of COVID-19 in Malawi. This account reveals shifting intersubjectivities of researchers and participants as the pandemic brought changes in the nature of the engagement, from ethnographic explorations into the roles of smoke in everyday life, through fieldwork suspension, and contextualised COVID-19 response. These experiences demonstrate how a basis of reflexive ethnographic engagement with communities can underpin thoughtful responses to upcoming challenges, with implications for future “global health” work, both within and beyond the pandemic context.

## Background: The Pamodzi Project

This paper presents personal reflections on an ethnographic endeavour in rural Malawi, and impacts of the COVID-19 pandemic on work in the village. In particular, I—as the main researcher (a female doctoral candidate from the United Kingdom with a background in public health)—describe and explore my position in the field and related intersubjectivities, as they developed throughout the ethnographic fieldwork, and subsequently with the advent of the pandemic.

This local, standalone ethnographic project called Pamodzi, meaning “together” in Chichewa (the main Malawian language): A title which reflects an aspiration for collaborative approaches, between researchers and community members. The project considered smoke—seen from “Western”, biomedical perspectives as air pollution—aiming to create an ethnographic account of this smoke (“*utsi*” in Chichewa) as experienced by residents of a single village in rural Malawi. This included smoke from cooking fires, as well as from other fires and alternative sources of combustion in residents’ lives. Extended periods of participant observation in households and public spaces in the village helped us to gain insights into how smoke was placed within residents’ wider concerns and priorities. In addition, bringing to the field what knowledge we had, in terms of the harmful longer term effects of air pollution on health, the project aimed to bring together perspectives on the issue, to deliver more contextualised insights into smoke in this setting. I also aimed then to work with participants in the village to co-develop potential “cleaner air” solutions, but this was left open to reimagining after the initial phase. Further details of the project and our epistemological approaches have been published elsewhere, in the main ethnographic paper and supplementary materials (Saleh et al., 2021).

The project started with our meeting a number of village chiefs and briefly discussing our ideas. We chose a village which was rural in nature, in keeping with most of the country, relatively near to where we were staying, and where the local chief and community health worker were supportive and interested in our project. The 8 month period of first-person participant observation which comprised the main body of the ethnographic project started with a period of walking around the village discussing initial research ideas and hearing individuals’ thoughts on our plans.

The mainstay of the participant observation involved accompanying household members (predominantly women) in their typical daily activities including food preparation, cooking, washing dishes, carrying water, and farming. This participant observation was done primarily by myself, along with male research assistant of Malawian nationality, who was employed through the research institution before the start of any fieldwork, and contributed much to the development of the research project. Our extended presence in the village allowed me to get a broader understanding of daily life in this setting, joining in with children’s (cooking-related) games and relaxing on straw mats with household members, chatting after a morning at the farm for instance, as well as participating in daily chores. A few months into the project, with the help of the assistant, I engaged a female member of the village community to work as a type of fieldworker, assisting in day-to-day arrangements and providing closer links with residents across the whole village.

Concurrent air quality monitoring involving the use of individual air quality monitors worn in waist bags allowed us to quantify levels of airborne particular matter throughout these activities, adding another perspective—this time more biomedical—to the account. In addition, face-to-face interviews with household heads and a series of six participatory workshops involving researchers and village residents allowed the inclusion of additional thoughts around smoke and created opportunities for now-engaged participants from the village to share thoughts around achieving cleaner air.

The epistemological approach of this transnational research project emphasised the recognition and valuing of participants’ knowledges alongside those of researchers (Biehl, 2016), with shared understandings developing through the research process.

## Wider Context: Traditional “Global Health” Approaches

The field of “global health” inherits a legacy of problematic ethical practice and researcher-participant relations nested in imperialist transnational relations (Greene et al., 2013). In research terms this is also played out in the academic imperialism which had asserted control over the relative valuing of knowledges, the extractive nature of knowledge production, and the presentation and dissemination of the academic product in global health research to date (Hountondji, 1997; Abimbola, 2019; Abimbola and Pai, 2020). Whilst these matters may initially seem conceptually far from the “field,” in terms of the Malawian village where the ethnographic project was based, overarching colonial legacies and enduring economic relations continue to touch individuals’ daily lives here in myriad ways (Saleh et al., 2021). The influences of these complex historical and political factors were evident from the start of the research project, with further developments in response to the advent of the COVID-19 epidemic in Malawi.

## Researcher Positionality

I am a female British researcher in public health (and clinician by background), trained in the United Kingdom. I am an independent researcher (a Wellcome Trust Clinical Fellow) but the research project was conducted under the umbrella of the Malawi-Liverpool Wellcome Trust in Blantyre, Malawi. I had no prior experience living in any African country and, whilst I am not typically “white” (I am of Iranian descent), my appearance and the way I am seen, partly in view of my childhood/early adulthood in the United Kingdom, have led to my experiences in the world being similar to those of a white person, with little exposure to explicit or implicit racism for instance. Similarly, my perspectives—shaped by “Western” formal education and socialisation—reflect British approaches to such topics as identity, society and health.

In the village, I was cognisant of how elements of my training lent me a “public health perspective,” in terms of my conceptualisation of issues relating to health and wellbeing of individuals and communities. This included my understanding of smoke, in terms of a barrier to “clean air” (and therefore to bodily health), although I understood this as one element of a wider social and political environment. Western medical influences, albeit tempered by anthropology training and relativist elements of my research approach, also shaped my understandings around other issues arising in the field, for example, episodes of illness in participants.

A final element of pre-existing positionality relates to my motivations for undertaking the research. As a public health researcher, my interest in the subject of “air pollution” had undeniably started from an awareness of its health effects, but—partly in response to seeing more typical clinical research approaches stemming from high-income institutions—I sought to understand this pollution (or “smoke,” as it was locally experienced) in its wider social, cultural and historical context. The choice of research site thus also linked back to previous research projects in similar settings (Mortimer et al., 2017). The ethnographic approach for me was important in providing the desired insights, in contrast to the more common behavioural and technological intervention studies in the field of air pollution research, in an aim to make any recommendations as relevant as possible to individuals’ lives in the village. Essentially, this related to a belief in justice in Global Health, rather than an attempt to help others as an act of charity (Abimbola, 2021). This remained an important motivator throughout the trajectory of the research period.

## Researchers’ Arrival in the Field and Development of Relationships During the Pamodzi Project

My roles and relationships within the ethnographic “field” developed throughout the research period and beyond and were subject to iterative reflection by myself and in discussion with the research assistant and local fieldworker, with episodic discussions also with qualitative senior researchers in the supervisory team helping to expand these reflective considerations. Perhaps the most immediately apparent aspect of the relationship at the outset of the project was economic: As a relatively light-skinned British person in the village, I was immediately recognisable as an “outsider” and thus was often asked, directly or indirectly, for money. This was perhaps inevitable in the context of extreme scarcity and transnational inequity in our contrasting backgrounds. The colonial histories of the United Kingdom and Malawi, and subsequent neocolonial relations, also undoubtably overshadowed relationships in terms of power differentials, above and beyond purely economic factors.

The significance of these dynamics was striking when compared to the influence of gender in the village. During the time spent with women in households, while there was occasionally a sense of female kinship—in discussions of our children for example—the difference in our lived experiences (together with my struggles with language) dominated. This meant that, in practice, despite the gender aspects, my research assistant felt more of an overarching shared identity with female residents in the village than me. This was an interesting indication of the magnitude of experiential difference between myself and participants in the village, and testament to my research assistant’s skill in expertly bridging the gap.

A further element of residents’ initial responses was a preformed expectation amongst residents of the form the project would take—stemming from experiences of similar transnational (health) research projects. This widespread narrative involved a description of an existing problem, to be solved by visiting “expert” research teams (usually by imported solutions, often involving some level of “technology”). On our arrival in the village, researchers and the project itself were seen in these terms—with village residents often asking questions around what we were bringing, introducing or teaching them—and it was not until a few weeks into our initial discussions and participant observations when residents’ understandings began to break away from this.

The balance of power in our ongoing relationships was nuanced and dynamic, however. Throughout much of the participant observation, residents in focused participant observation households were relatively “at ease”, assuming a role akin to teachers or guides, in relation to routine activities in the village. Throughout these activities, I was the only non-Malawian person present, equipped with limited Chichewa or cultural knowledge, but a perpetual eagerness to learn. The possibility of prolonged physical presence in the field, often involving a vulnerability for me in particular, as when I attended a coming-of-age ceremony (“*chinamwali*”) restricted to women, contributed to an enduring sense of trust within these relationships. This was apparent from the regular invitations into households to participate in daily activities, to special occasions, jokes, conversations and even ad hoc dancing amongst women in excited conversations about upcoming ceremonies for instance.

Researcher-participant dynamics were made more complex by the introduction of additional methodologies, for instance where participants were asked to wear air quality monitors, and when recorded interviews took place and the traces then displayed on a screen and discussed with participants. This highlighted the shared learning emerging from the project, as we heard about local smoke-producing practices, and participants’ responses to smoke, whilst bringing new ways of quantifying smoke levels. Local wisdom around fuel use and place of cooking did take into account avoidance of smoke to an extent for instance, although much of our learning was also around the wider restrictions in lived experience which often made longer-term health concerns relating to smoke a secondary consideration (Saleh et al., 2021).

Participatory workshops—held in Chichewa by an external facilitator—further challenged the assumptions around power within relationships as researchers took part in games, exercises and discussions alongside residents. A careful use of methods here aimed for a community of co-learning and questioning, creating new spaces for participation and discovery (Etmanski, 2014). This lent the entire ethnographic period a sense of constantly shifting intersubjectivities which served to destabilise any unilateral power imbalance.

## Initial Responses to the Threat of COVID-19—Ending Ethnographic Fieldwork

The advent of COVID-19 was at a time when ethnographic activities were naturally receding, with only a few final air quality monitoring activities and some general village discussions ongoing. I was very aware of the likelihood of the SARS-CoV-2 virus (COVID-19) being introduced to Malawi through the geographical mobility of economically privileged groups (particularly to/from continents with already high numbers of cases, such as Europe). This awareness, together with our now-warm relationships with many extended families throughout the village, left me thinking hard about ways to protect village residents from the threat of COVID-19. In particular, partly influenced by my medical grounding, I thought of older members of the community who would likely be at increased risk from the disease, and of the challenges in accessing clinical care for people in the village setting.

My first action as project leader, therefore, was to start drawing study activities in the village to a close, explaining to participants what we knew of the nature of COVID-19 and about our plans to stop the fieldwork. We were clear that we as researchers did not want to be a source of possible disease, thus moving away physically at this time—whilst sad in many ways—constituted an important protective action. This was the start of an emergence of different intersubjectivities in our research relationships, as my medical and “public health”-related positionalities shaped responses to the unfolding pandemic.

Although we had in any case been approaching an end to the fieldwork, the decision to stop under these conditions brought unique challenges. The decision having been made at short notice, and in response to the pandemic, was relatively unplanned, and thus felt imposed upon us rather than being co-developed in the field, as many other aspects of the work had been. Persisting unknown elements relating to COVID-19 added to this difficulty, accentuating the sense of general uncertainty which often accompanies the process of “leaving the field” (Iversen, 2009). Lastly, village-wide activities which we would have carried out to mark this point of (albeit temporary) closure, express our gratitude to village residents for welcoming us, and discuss some of our findings, were not possible due to COVID-19 precautions. Instead these functions had to be fulfilled through ad hoc conversations with individuals and small groups, alongside explanations of why physical contact (such as shaking hands) was being restricted and so on^1^. This divergence in concern around COVID-19, with the pause in activities being driven by us as researchers, again accentuated the differences in researcher-participant positionalities, and concurrent issues of power in terms of decisionmaking around the project.

A key difference here between our “leaving the field” and accounts of this in the literature—and indeed between what we had expected at this point—lay in the nature of our “leaving” (Iversen, 2009; Michailova et al., 2014; Fitzpatrick, 2019). In the present case, while our daily physical presence as ethnographers was coming to a close, our engagement in the field continued, albeit with a shift in terms of roles. This lack of finality made the process more complex, but highlighted the processural nature of ethnographic exit, rather than a one-off event. This process, as suggested by Michailova et al. (2014), was invaluable in stimulating the current exploration of researcher identities and intersubjectivities in the field.

Further to this physical withdrawal, drawing on my background as a public health doctor, and building on what we knew about the village setting, we went beyond simple “health education” activities, taking steps to ensure that residents would be able to put into practice common “COVID-19 prevention” recommendations after we left. We provided soap and buckets to allow handwashing at key points in the village and, later, cloth facemasks for residents. These developments shifted our positionings in the village, with a role more weighted towards bringing external knowledge and resources to residents in the village, and positioned me more explicitly in my prior “public health” role.

In view of the nature of researcher-community dynamics fostered thus far, involving collaborative integration of local and external knowledge, the ethnographic project felt resilient to this challenge. Residents themselves did not feel COVID-19 to be a relevant issue in their lives. Thus, while residents were respectful and appreciative of the support, with handwashing stations displayed at prominent points in the village for example, the trust built in many relationships permitted a degree of candidness not often seen in more formal relationships in Malawi. This was evident particularly in the gentle joking around the threat to us—“people coming from town”—posed by COVID-19.

The step away from in-person fieldwork (and review of the ethnographic fieldnotes at this time) highlighted the privilege which had been afforded to us as ethnographers (my research assistant and I) in previous months. Our physical presence within the village community during this time, laying the groundwork for trusting relationships, had allowed us to better understand how “smoke” fitted within the wider environment of individuals’ daily lives in and around the village. In the months following the suspension of our ethnographic fieldwork, my research assistant and I were often asked—on sporadic visits to the village for other purposes—why we were no longer present. On hearing our response, the usual reply was “but there isn’t any COVID-19 here”. Whilst highlighting communities’ perceptions of COVID-19, this also suggested that residents did not consider us as researchers to be bringing risk of disease, as may have been the case. Nevertheless, the pause in ethnographic fieldwork continued, and other more traditional “public health” activities temporarily took its place.

## COVID-19 in Malawi: Further “Public Health” Responses

In the ensuing weeks, numbers of COVID-19 cases in Malawi slowly climbed, and perceptions of village residents were substantiated as cases were mainly seen in the city. In more systematic considerations of how rural communities in Malawi (constituting a large majority of the country) may be protected as far as possible from the spread of the disease (The World Bank, 2020), my original “public health”-positionality again came to the fore. At this point, my research assistant and I became involved in a local COVID-19 response project: “*Kuteteza*” (meaning “to protect” in the local language, Chichewa). Kuteteza involved supported “shielding” of consenting residents over 60 years of age: Household-level arrangements were made to allow these older individuals to stay alone, minimising their external social contacts, and to be supported in chores such as collection of water, food, or cooking fuel. The small amounts of funding available covered additional soap, handwashing buckets and masks for involved households. The village in which our ethnographic project had been based was one of the first to be introduced to this project. We approached the local chief and other gatekeepers personally in the first instance to discuss the approaches, although the mainstay of the project employed local Health Surveillance Assistants and local volunteers for implementation.

While maintaining engagement with communities and a voluntary approach to participation throughout, the project still represented a further departure from the aims and attitudes espoused by the ethnographic research project. The introductory discussions and provision of information reinforced a slight shift in our positions in the village, with the foregrounding of our imported knowledge, and suggestions of relevant responses for residents to take. The handover of day-to-day operations in the project to local staff allowed us to step away from this role however, and it subsequently appeared that the less formal, somewhat closer relations forged throughout the ethnographic period had to an extent endured. Key contacts in the village sporadically contacted us informally through text- and social media messages, in addition to the regular situational updates, asking when we might return. The continued uncertainty around the national epidemic made project planning—and therefore responses to these enquiries—difficult, and again highlighted the source of decisions as external to the village, rather than participant-driven.

Once the first wave of COVID-19 had subsided, the project was able to recommence, with small, outdoor village meetings, and the introduction of locally made cookstoves for cooking. Evaluation of these was largely ethnographic in nature, involving walks around the village and ad hoc conversations with residents, with whom we were now familiar. Whilst in-depth discussion of this third “phase” of activity (following initial ethnographic work, then COVID-related activities) lies outside the scope of the current paper, it is relevant to note how that this did allow a return to our original project considerations. The sense of our making a contribution to village life with the stoves—which were very well-received—was made clear by participants around the village. This felt significant to me after the generous welcome and patience we had experienced from so many participants throughout the project. Our presence also allowed a more formal conclusion to the project, and hence a sense of closure for all involved.

## COVID-19 in Malawi—Perceptions and Experiences in the Village

In Malawi, the direct impact of the COVID-19 epidemic continued to be felt mostly in urban areas. Newspaper reports and local conversations highlighted deaths of statesmen, pastors, and other members of the urban “elite” (Chimjeka, 2021; Kasanda, 2021; Matonga, 2021; Mwanjansi Mwakikunga, 2021). Further, for reasons that remain unclear (Chibwana et al., 2020), numbers of cases, particularly in the first wave (around June to August 2020), were much lower than expected by many in the global community (RCO Malawi, 2021; United Purpose, 2021).

In the village we observed this first-hand: Through messages and phone calls, and on occasional careful visits over the following months, it became clear that local perceptions of COVID-19 remained largely unchanged. Accounts from residents, including the chief, painted a picture of few if any identified cases of COVID-19-related illness or death and no noticeable change in local morbidity or mortality rates. Village residents often described COVID-19 as “matenda a ku towni” (disease of the town), or even “matenda a anthu okudya za mafuta” (disease of people who eat relish containing oil, ie., rich people). For members of the village community who encountered ample risks and hardships in the course of their daily lives, it seemed that the threat posed by this disease was more of a theoretical one than a fear rooted in lived experience (Saleh et al., 2021). This echoed in some ways residents’ initial responses to the idea of smoke as a potential source of harm, which was introduced in the original project.

COVID-19 did not however leave residents’ lives untouched. Indirect impacts of the epidemic included increased transport costs due to social distancing on minibuses and fuel prices; and expenditure on masks, required for use in government facilities for example. For many, healthcare access was also impacted as widespread rumours, largely relating to the intentional spread of COVID-19 by healthcare professionals, created fear around government health facilities and they turned instead to private clinics, or stopped attending formal healthcare settings altogether [E. Makepeace (researcher and consultant), written communication, March 2021]. Nevertheless, a common perspective from those living in the village remained one of sympathy for us as “town dwellers,” whose lives seemed to have been more appreciably impacted by the epidemic.

In the light of this, it was striking to see international coverage, particularly from non-governmental organisations, which tended towards conventional narratives of Africa as a site of suffering and of African bodies as needing salvation from the West. These reports persisted even when the classic overwhelmed health systems and “bodies on the streets” did not transpire. Again, we were witness to how legacies of transnational relationships persisted in the global imagination (Chikaonda, 2020).

Through the window of the ethnographic project in Malawi and beyond, through its official suspension, I gained insights into the enduring legacies of colonial and post-colonial transnational relations for research relationships such as ours. In this case, the COVID-19 pandemic illuminated the ways in which these historically grounded “global health” relations persist in shaping perspectives on and responses to new global health challenges.

## Discussion

In this paper I have attempted to provide an account of changing roles and relationships in the field, influenced by the advent of COVID-19. This reveals how our presence in the village as researchers (over the prior months of in-person participant observation), helped us to develop robust relationships, allowing for meaningful, engaged “public health” responses to COVID-19, when it arrived. From our arrival in the village, it was clear that national and institutional colonial histories played a part in shaping how we were seen on our arrival in the village. Conversely, the COVID-19 pandemic soon revealed how colonial relations continue to frame popular perceptions of Malawi in the “Western” imagination as a site of suffering and dependency. Extended in-person ethnographic experience in this case revealed alternative perspectives based on real experiences in “the field”.

A key aspect of ethnographic fieldwork involved elements of embodiment running through my immersion in daily activities alongside female residents. Various elements of my position as an outsider in this context—as a researcher from a Western background, with entirely different life experiences to this point—clearly restricted my access to “a complete understanding of participants” lived experiences.

Acknowledging this, the embodied experience of cooking nsima on a three stone fire, as well as various other daily activities, permitted what has been described as a “a sensory engagement with others’ lifeworlds and lived realities” (22, p.21). Husserl’s concept of intersubjectivity is relevant here, relating to the *possibility* of being in another’s place: of understanding another’s perspective (Husserl, 1989). This critical facet of ethnography could not have been achieved through other qualitative approaches such as interviews or focus group discussions. As women schooled me in turning the thick nsima without pausing, despite the smoke making my eyes and nose run and splashes of the boiling mixture hitting my bare feet, shared understandings also forged a deep rapport transcending those typically seen when alternative modes of research are employed (Okely, 2007).

While it has been posited that these processes can lead to a dissolution of the power differentials between the ethnographers and their participants, bringing them to an equal footing (Kesselring, 2015), for me this represents a claim too far. The arrangement of historical and structural factors shaping my position in the field could never be dispersed so quickly or completely, but what transpired was a sense of a changed relationship emerging from these accumulating encounters. With the advent of the COVID-19 pandemic, the repercussions of these relationships, stretching beyond the period of my presence in the ethnographic role, became clear.

In subsequent months, in redirecting energies towards responding to the pandemic threat, I found that, alongside elements of my original “public health” perspective such as traditional scientific evidence on COVID-19 transmission, the deep understanding of residents’ lived experience helped to inform the suggestions brought to the village. These relationships with residents allowed us insights into their perceptions around COVID-19 and paved the way for them to provide us with honest feedback on our attempts to help. This dynamic echoed the initial project aspirations, as I aimed to bring my clinical and scientific knowledge into conversation with participants’ situated knowledges.

Similar roles in medical anthropology have been identified and put into practice by social scientists involved in the Ebola response in West Africa (Stellmach et al., 2018). The Ebola Response Anthropology Platform (ERAP), and subsequently the Social Science in Humanitarian Action Platform (SSHAP), share examples of how local and international social scientists have contributed to context-sensitive, and thus more engaged and effective crisis responses in various low resource settings (Social Science for Emerge, 2021; IDS U et al., 2021. This includes attention to local practices such as care of the sick and burial in epidemic settings.

Melissa Leach, one of the anthropologists involved in creating ERAP, highlighted in a recent publication the need for ethnographers to reflect on their own positionalities and approaches to their field sites (Leach, 2019). In interrogating my history as a Western (British) researcher entering Malawi, I have been aware of colonial histories, and of the role of anthropology—at times—in supporting the colonial project (Malinowski, 1929; Asad, 1973). The parallel troubled histories of colonial “tropical medicine” in countries such as Malawi (Vaughan, 1992; Hokkanen, 2019), give ample cause for reflection on the relationships with power that I carry with me on my approach to the ethnographic “field”.

The complexity of these legacies and roles in anthropology are also important, however. From early in its history, key figures in anthropology have spoken against its deployment towards imperialist aims (Boas, 2005). In a subtler sense, we are witnessing current applications of anthropological insight in “global health” projects introducing shifts in perspective: moving beyond colonially-inspired narratives—of “donor-recipient”-type relationships, for example—and toward a more connected presence within communities. This can in turn create increasingly balanced and thoughtful contributions to global health endeavors.

A final point on this piece is to note that this reflective account is limited to my personal experiences. It does not speak to the experiences of other members of the research team, and could be said to underrepresent study participants’ own perspectives, thus echoing the ongoing imbalance in Global Health research. Proposed solutions to this include the use of participatory research approaches and changes to the academic systems (incorporating funding, publishing and so on) to reduce barriers to academic involvement and representation for low- and middle-income country actors themselves (Baum et al., 2006; Wallerstein et al., 2019; Busse and August, 2020; The Lancet Global Health, 2021).

## Conclusion

Ethnography is unique and relatively uncommonly utilised in comparison with other qualitative research methods. The ethnographic researcher is afforded the privilege of broad and in-depth insights into communities’ daily lives, values and priorities. Participant observation methodologies involve intricate participant-researcher relationships, with the historical and geographical elements of many “Global Health” research projects introducing further complexity.

The experiences outlined in this piece illustrate how the practice of ethnographic fieldwork created understandings and relationships which influenced the subsequently devised public health responses to the COVID-19 pandemic when it arose. Whilst navigating developing relationships in the field and my own positionalities was at times challenging, prior experiences conducting ethnography proved invaluable in planning and carrying out contextually relevant and appropriate pandemic responses.

## Data Availability

The raw data supporting this article, in the form of field notes and reflective notes, cannot be made available due to the personal and confidential nature of the content, and risks of compromising participant confidentiality.

## Footnotes

1 Residents had in fact heard about COVID-19 at this point from the government messages on the radio, and through social media and word of mouth. They were therefore were aware of preventative measures, which were also being introduced nationally. As a result, although we took care to explain our actions and the reasoning behind them, these did not come as a surprise to participants, in the context of wider events.
